# World-wide survey on the treatment of peripheral vestibular disorders

**DOI:** 10.3389/fneur.2025.1540443

**Published:** 2025-02-04

**Authors:** Michael Strupp, Nils Lucca Kern, Göran Laurell, Louisa Lehner, Eva Grill, Ralf Strobl

**Affiliations:** ^1^German Center for Vertigo and Balance Disorders, LMU University Hospital, LMU Munich, Munich, Germany; ^2^Department of Neurology, LMU University Hospital, LMU Munich, Munich, Germany; ^3^Department of Surgical Sciences, Uppsala University, Uppsala, Sweden; ^4^Institute for Medical Information Processing Biometrics and Epidemiology, LMU Munich, Munich, Germany

**Keywords:** peripheral vestibular disorders, BPPV, acute unilateral vestibulopathy, Menière’s disease, bilateral vestibulopathy, vestibular paroxysmia, SCDS, betahistine

## Abstract

**Objective:**

The aim of this world-wide survey was to evaluate the currently applied treatment options for the six most frequent peripheral vestibular disorders: benign paroxysmal positional vertigo (BPPV), acute unilateral vestibulopathy (AUVP)/vestibular neuritis, Menière’s disease (MD), bilateral vestibulopathy (BVP), vestibular paroxysmia (VP) and superior canal dehiscence syndrome (SCDS).

**Background:**

For the therapy of vestibular disorders, there are four treatment options: vestibular physical therapy (canalith repositioning maneuvers or balance training), pharmacotherapy, surgery, and psychotherapy. Since there are very few state-of-the-art RCTs, the treatment of vestibular disorders is so far not standardized and various methods are applied with heterogeneous efficacy.

**Design/methods:**

A web-based standardized survey questionnaire on the treatment of the six most frequent peripheral vestibular disorders was used to collect data.

**Results:**

234 replies from five continents, 47 countries, 162 cities and 188 centers were received: (% from all 234 replies; multiple answers possible): BPPV: posterior canal BPPV: 71% Epley, 40% Semont, and 12% others. Horizontal canal BPPV canalolithiasis: 58% Lempert (roll-over) maneuver, 33% Gufoni, 7% prolonged rest, and 9% others. Horizontal canal BPPV cupulolithiasis: 35% Gufoni, 27% Lempert (roll-over) maneuver, 9% Zuma, and 7% head shaking: AUVP: 79% pharmacotherapy, namely 47% glucocorticoids, 39% antiemetics, and 24% betahistine; 67% vestibular physical therapy. MD: 85% pharmacotherapy, namely 65% betahistine, 21% diuretics, 20% steroids, 16% antiemetics, 14% gentamicin; 37% surgery. VP: 65% pharmacotherapy, namely 57% anticonvulsants; 7% surgery. BVP: 77% vestibular physical therapy. SCDS: 50% surgery, namely 38.8% canal plugging, 23.3% capping and 15.5% resurfacing.

**Conclusion:**

In this world-wide survey with 234 replies from 188 centers, widely heterogeneous applied treatment options were reported for the six most frequent peripheral vestibular disorders. This study shows in particular that certain drugs are often used despite low or very low evidence. Namely in AUVP, MD and VP well-designed controlled trials with clinically meaningful endpoints are needed.

## 1 Introduction

Peripheral vestibular disorders are a common medical issue (1). The most frequent peripheral vestibular disorders are Menière’s disease (MD), acute unilateral vestibulopathy (AUVP), benign paroxysmal positional vertigo (BPPV), bilateral vestibulopathy (BVP), vestibular paroxysmia (VP) and superior canal dehiscence syndrome (SCDS). The main therapeutic approaches consist of vestibular physical therapy (canalith repositioning maneuvers or vestibular exercises, balance training and gait training), pharmacotherapy, surgery and psychotherapy. There is no standardized treatment of patients with peripheral vestibular disorders, mainly because state-of-the-art placebo-controlled trials on therapeutic measures are lacking. In clinical practice, different approaches and their combination are used, based mainly on joint expert opinion and not on standardized treatment protocols. The lack of comparable randomized controlled trials (RCT) might be due to a shortage of consensus regarding the optimal methodology for conducting such studies. This warrants further attention as therapeutic recommendations can only be meaningfully derived if studies are comparable and conducted to the same measures. Lacking recommendations on a clear and concise treatment strategy in peripheral vestibular disorders lead to a non-standardized, diffuse therapy in daily clinical practice. This can have a serious and significant impact on patient health and the success of therapy. To further address this problem, a standardized world-wide web-based survey, supported by the Bárány Society, was conducted on the quantitative and qualitative real-world treatment of the six most frequent peripheral vestibular disorders. The results of this survey may provide guidance for therapeutic approaches and successful symptomatic treatment with possible effects on daily clinical practice.

## 2 Methods

### 2.1 Data collection procedures and participants

Data was acquired cross-sectionally by means of a web-based standardized survey questionnaire conducted between August 2020 and March 2022. To maintain a level of expertise in peripheral vestibular disorders potential participants were identified via the network of the Department of Neurology and German Center for Vertigo and Balance Disorders of Ludwig-Maximilians University Munich as well as the Bárány Society. An invitation was sent to colleagues from all countries in the world. All data was collected online using the SoSciSurvey tool [SoSci Survey (Version 3.1.06); computer program] (2019).^1^ All analyses were conducted using R (R Core Team. R: A language and environment for statistical computing. R Foundation for Statistical Computing, Vienna, Austria; 2022. Version 4.2.2).

### 2.2 Standard protocol approvals, registrations and patient consents

The questionnaire was filled out by physicians. Neither patients nor healthy controls were directly included, thus neither an approval nor a registration or patients’ consents were required for this survey. As this study is an anonymized qualitative study of health experts an ethics approval was not necessary. Specifically, the survey was conducted using SoSciSurvey tool, a platform compliant with GDPR standards and designed to uphold participant confidentiality. Responses were collected anonymously, ensuring that no identifying information was linked to individual participants.

### 2.3 Questionnaire items

The online survey questionnaire consisted of 1,272 items, including questions on the participating respondent, such as the residing center, the experience in the field, the approximate number of patients with peripheral vestibular disorders seen per year and the basic symptomatic treatment method for patients with vertigo and dizziness. Four basic treatment options for peripheral vestibular disorders were examined in detail: (1) vestibular physical therapy, (2) pharmacotherapy, (3) psychotherapy and (4) surgery.

Regarding vestibular physical therapy, the questionnaire asked about applied canalith repositioning maneuvers, including frequency of their application and duration (assessed in days), the performing person as well as potential follow-up examinations and their timing. Furthermore, respondents were asked if other physical therapeutic approaches were used, such as vestibular exercises, balance or gait training. If so, frequency, duration and the performing person was requested.

If pharmacotherapy was used, the name of the medication, the dosage form (oral or intravenous), the total daily dosage (mg per day) and the duration of treatment was assessed. For pcBPPV, acBPPV and hcBPPV the use of vitamin D with its dosage and duration of treatment was of interest.

If patients received psychotherapy, participants were asked to provide information on type, duration, and the executing healthcare professional (doctor or psychotherapist).

For surgery, information on the main diagnosis, the performed procedure and the time latency from onset of symptoms until surgical treatment was assessed.

The questionnaire was available in English only. Answers could be given as open text, dichotomous or via checkbox. Multiple responses were possible. The participants were presented with several therapeutic options, and therefore their response was counted multiple times. For example, in the pharmacotherapeutic approach, multiple pharmaceutical agents applied could be reported. The full survey questionnaire can be found in the supplementary materials.

### 2.4 Statistical analysis

Descriptive statistical analysis was performed with Microsoft Excel and R (R Core Team. R: A language and environment for statistical computing. R Foundation for Statistical Computing, Vienna, Austria; 2022. Version 4.2.2). Statistical analysis was carried out using Microsoft Excel and R. Categorical data are expressed as numbers with percentages and continuous values are expressed as median and range. The percentages shown are referenced to all 234 replies.

## 3 Results

**Figure 1:**
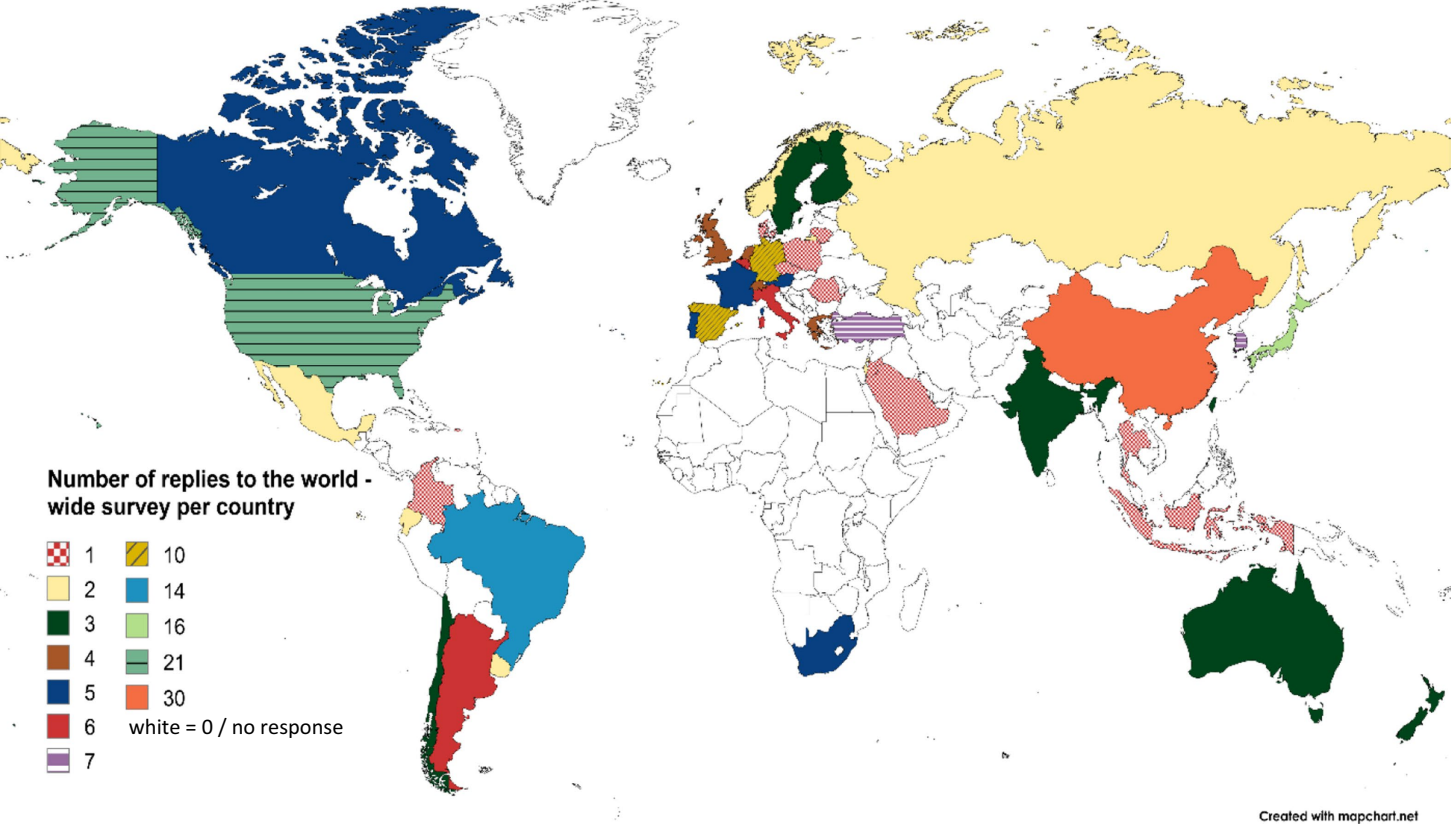
Number and geographical distribution of centers that replied to the world-wide survey questionnaire. Created with mapchart.net.

Between August 2020 and March 2022, 234 questionnaires from five continents, 47 countries, 162 cities and 188 centers were received. Participating countries can be seen in Figure 1.

### 3.1 Menière’s disease

**Figure 2:**
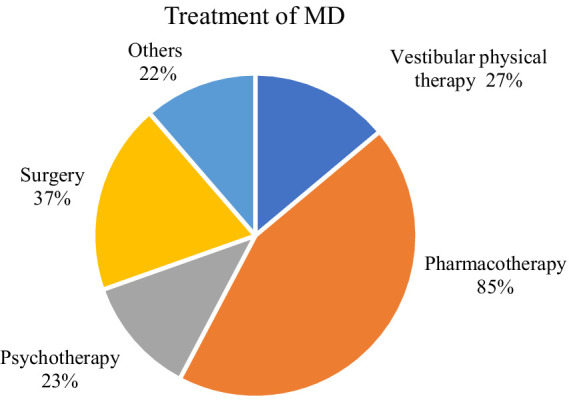
Treatment options in MD. Created with mapchart.net.

**Figure 3:**
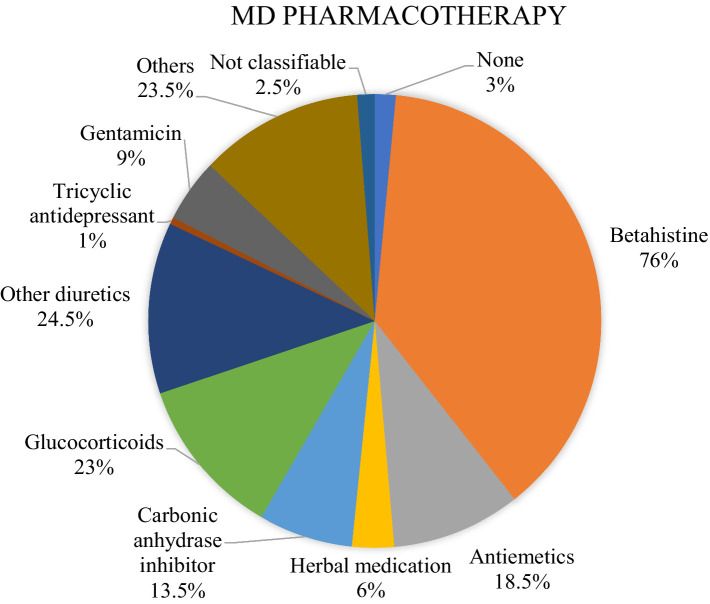
Overview of the medication used in MD.

In Menière’s disease, vestibular physical therapy in the form of vestibular rehabilitation (e.g., balance training) was used by 27% of physicians. Most MD patients were treated with pharmacotherapy (85%; Figure 2). With 76% betahistine was the most frequently used medication followed by diuretics with 24.5% and glucocorticoids with 23%. 18.5% received antiemetics (antihistamines, dopamine antagonists, serotonine agonists), 13.5% carbonic anhydrase inhibitors, 9% gentamicin, 6% herbal medication and 1% tricyclic antidepressants (Figure 3). Surgery was reported by 37% of the participants. The most common procedure was endolymphatic sac decompression with 47.1% followed by intratympanic injection of gentamicin (37.9%) as a minor surgery. Labyrinthectomy was performed in 25.3%. Vestibular neurectomy and intratympanic steroid injection (minor surgery) were reported with an equal frequency of 20.7%. The least frequent applied surgical method was canal occlusion with 14.9%. 23% of respondents treated their patients with psychotherapy, with 36% cognitive behavioral therapy (CBT) was mainly used. The most frequent combined treatment approaches were pharmacotherapy and surgery (21.7%) followed by combining pharmacotherapy, vestibular physical therapy, surgery and psychotherapy (10.8%).

### 3.2 Acute unilateral vestibulopathy

**Figure 4:**
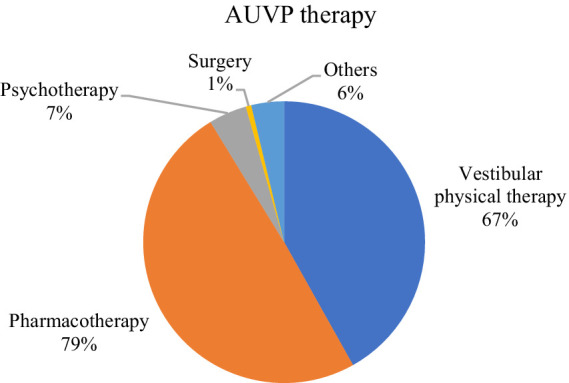
Treatment options in AUVP.

In patients with AUVP, the most common treatment strategy was pharmacotherapy with 79% (Figure 4). Vestibular physical therapy was the second most common treatment strategy with 67%. The most frequent applied combination of treatment approaches was pharmacotherapy and vestibular physical therapy. The vestibular physical therapeutic approach consisted mainly of vestibular rehabilitation (e.g., balance training; 41%). Glucocorticoids were the main drugs used (59.8%). 50% received antiemetics, 29.9% betahistine. 40% of the respondents reported a combination of a pharmacotherapeutic approach with corticosteroids and a vestibular physical therapeutic approach. Alternative therapeutic approaches like herbal medication were also used; the detailed distribution can be seen in Figure 4. Medications were usually given for 1–7 days (49%). 15% administered medication for 8–14 days, 17% for 15–30 days, 11% for 31–90 days and 1% for 91–180 days. Medication was most frequently administered orally (38%), followed by intravenous (24%) and intramuscular injection (10%). As there is no indication for a surgical approach none was reported. A small number was treated with psychotherapy (7%), with CBT again being the most used therapy.

### 3.3 Benign paroxysmal positional vertigo

In BPPV, the participants were asked for their therapeutic strategies depending on the affected canal. Regardless of the affected semicircular canal, vestibular physical therapy was the most used therapeutic approach.

In posterior canal BPPV (pc BPPV), the Epley maneuver was used by 91.2%. 51.4% used the Semont maneuver, 8.3% Brandt-Daroff exercises. Other maneuvers (detailed overview can be seen in Table 1) were used by 25%. 15% of the respondents stated that they also used drug therapy in pcBPPV patients. Of those 58% used betahistine, 27.8% antiemetics, 19.4% Vitamin D and 11.1% each calcium channel blocker and herbal medication. Vitamin D was mostly administered in a daily dose of 1,000 IU or above, 57% of participants applying Vitamin D reported a treatment duration of more than 3 months (Table 2). 9% reported surgical therapy, the main surgical treatment approach was a canal plugging of the posterior semicircular canal (70%). Psychotherapy was among the least frequent treatment approaches (5%). A follow-up examination was reported only by half of the participants (55%), 39.5% managed to see their patients within 3–7 days.

**Table 1:** Vestibular physical therapy in BPPV—overview of used canalith repositioning maneuvers in pc BPPV and hc BPPV—can.

Pc BPPV—vestibular physical therapy (*n* = 181)	Hc BPPV—can—vestibular physical therapy (*n* = 174)
Epley 165 (91.2%)	Lempert (roll-over)136 (78.2%)
Semont 93 (51.4%)	Forced prolonged positioning 17 (9.8%)
Brandt-Daroff 15 (8.3%)	Gufoni 77 (44.3%)
Others 28 (15.5%)	Zuma 7 (4.0%) Appiani 3 (1.7%)
	Vanucchi-Asprella 4 (2.3%)

In horizontal canal benign paroxysmal positional vertigo (hc-BPPV), respondents were asked to differentiate between canalolithiasis (hc-BPPV-can) and cupulolithiasis (hc-BPPV-cup). In hc-BPPV-can, vestibular physical therapy was the main treatment approach with its various canalith repositioning maneuvers (77%); the Lempert (roll-over) maneuver was used by 78.2%, Gufoni by 44.3% and forced prolonged positioning (Vanucchi) by 9.8% (Table 1). Pharmacotherapy was used by 14% of the respondents. Betahistine (66.7%), antiemetics (18.2%), herbal medication (18.2%) and vitamin D (12.1%) were the most common. Surgical and psychotherapeutic measures were secondary (5% each). In hc-BPPV-cup, the main treatment strategy also consisted of vestibular physical therapy in the form of canalith repositioning maneuvers (69%). The most commonly used was Gufoni (50%) followed by the Lempert (roll-over) maneuver (38.3%), Zuma (13.6%) and head shaking (9.9%). Analogous to hc-BPPV-can, pharmacotherapy was used by 15% in hc-BPPV-cup. Drug therapy was dominated by betahistine (69.4%). Antiemetics (22.2%), herbal medication mainly gingko drugs (11.1%) and vitamin D (11.1%) were used by few. Nine surgical approaches (3.8%) via canal occlusion were reported. Psychotherapy was used by none of the participants (Table 2).

**Table 2:** Vit. D in pc BPPV—duration in months and dosage in IU.

Duration of intake in months (n)	Vitamin D dosage IU (n)
None (0)	None (2)
1 (3)	0–1,000 (5)
2 (1)	> 1,000 (7)
3 (4)	Not classifiable (5)
> 3 (3)	
Not classifiable (2)	

Anterior canal benign paroxysmal positional vertigo (ac BPPV) was mainly treated by vestibular physical therapeutic maneuvers as well (57%, 133 physicians); the Yacovino maneuver was performed in 62.4% and the modified Epley maneuver in 24.1%. Only 10% used pharmacotherapy, betahistine was the main reported drug (60.9%). Three participants reported on vitamin D treatment. Surgery was reported by none of the respondents. Psychotherapy was used in 2%, the answers were classified as “not classifiable.”

### 3.4 Bilateral vestibulopathy

The main therapeutic approach in BVP was vestibular physical therapy (77%); vestibular rehabilitation training (29%) in the form of balance training (37%) was mostly used. We received feedback from 45 participants (19%) stating that pharmacotherapy was also used, mainly betahistine (55.6%). Four physicians reported on surgical measures (2%), here vestibular implants were used after non-invasive therapy failure. 9% reported on psychotherapeutic treatment. Mainly CBT was applied (36%). The combination of pharmacotherapy and vestibular physical therapy was the most frequent applied treatment combination (14.4%); 6.2% combined vestibular physical therapy and psychotherapy.

### 3.5 Vestibular paroxysmia

Pharmacotherapy was the main treatment strategy in VP (65%). Anti-seizure medication (ASM) was used in 88.7%. Of those, 75.3% were treated with carbamazepine, 35.8% with oxcarbazepine, 16.4% with gabapentin, 2.9% with lamotrigine, 1.5% with topiramate and 0.75% with levetiracetam. 7% reported using vestibular physical therapy in the form of vestibular rehabilitation (e.g., balance training). Surgery was reported by 9%; 77.3% used the Janetta-decompression method. Psychotherapy was used by 2.5%. Pharmacotherapy and surgery were the most common combined therapy approaches (9.5%).

### 3.6 Superior Canal dehiscence syndrome

We received feedback from 50% of the participants stating that surgery was the treatment of choice. 38.8% of those were treated by canal plugging, 23.3% by capping and 15.5% used resurfacing. 12% reported using pharmacotherapy, the following medications were primarily used: betahistine (37%), antiemetics (29.6%), carbonic anhydrase inhibitor (11.1%) and tricyclic antidepressants. Vestibular physical therapy was the therapeutic strategy in 9% of the participants. Psychotherapy was subordinate. Pharmacotherapy and surgery were the most frequent combined therapeutic approach (10.1%) followed by surgery and vestibular physical therapy (6.8%).

## 4 Discussion

This web-based standardized questionnaire focuses on the real-world treatment of the six most common peripheral vestibular disorders and the primary therapeutic approaches: vestibular physical therapy, pharmacotherapy, surgery, and psychotherapy. Our world-wide survey, which received 234 responses from 188 centers, suggests utilizing these treatment options either individually or in combination to address the above-mentioned disorders. As a result, the specific approach taken did vary, indicating that clear therapeutic guidelines are missing.

### 4.1 Menière’s disease

According to our world-wide survey, in MD the main therapeutic approach for preventive treatment was pharmacotherapy (85%) with betahistine being the most frequently orally applied drug (76%). Despite the routine use of betahistine in clinical practice, the evidence is very uncertain. This was demonstrated by a recently conducted Cochrane Review comparing seven randomized controlled trials (RCTs) (2). Due to the different primary outcomes and follow-up time points, there was no adequate basis for comparability so that no conclusions on the effect of betahistine could have been drawn. Other reported drugs in our survey were diuretics (24.5%) and glucocorticoids (23%). There are only two studies assessing the use of diuretics (each comparing different diuretics to placebo) (3, 4) and one study assessing the use of oral steroids (5), all with a very low-certainty evidence. For symptomatic treatment of episodes of MD, antiemetics (antihistamines, dopamine antagonists or serotonin agonists) are widely used (6), in our survey only 13.5% reported a usage though. Of those who reported a surgical treatment approach, 37.9% reported of the minor surgery intratympanic gentamicin injection and 20.7% of intratympanic corticosteroids. In recent Cochrane reviews, it was concluded that the evidence for both measures is very uncertain (7, 8). Our survey revealed that endolymphatic sac decompression was more frequently used than intratympanic injection (47.1% vs. 37.9%). This is surprising because the efficacy of this very invasive measure has not been proven by any study (9). Vestibular physical therapy was used by 27% of our respondents, consisting of vestibular rehabilitation in the form of balance training. There is heterogeneous data on the effects of vestibular physical therapy. A systematic review assessing the efficacy of vestibular rehabilitation showed no clear improvement on balance and dizziness related to quality of life in MD patients (10). Another systematic review on the effectiveness of vestibular rehabilitation in patients with symptomatic unilateral peripheral vestibular dysfunction revealed moderate to strong evidence that this therapeutic approach is safe and effective (11).

A missing consensus on the optimal treatment strategy is mirrored in our findings. The evidence for the efficacy of the various currently applied methods in MD is low or very low (2, 12–17). This is due to a lack of standardized RCTs with clinically relevant endpoints and explains the wide spectrum of measures taken by doctors all over the world. Especially the role of betahistine remains controversial: there is no evidence for its efficacy in dosages of up to 48 mg three times a day (18). However, theoretically the combination of betahistine with a monoaminoxidase B (MAO-B) inhibitor could be effective because with this approach 100 times higher plasma concentrations can be reached, leading to a high histamine 3 receptor occupancy (19). This can be beneficial due to its indirect activation of histamine 1 receptors which are found in high intensity in the inner ear (20) leading to an increased permeability of the intrastrial fluid-blood barrier. In conclusion, there is an urgent need for state-of-the art RCTs to improve the treatment of patients affected.

### 4.2 Acute unilateral vestibulopathy

The treatment in AUVP is based on three principles: symptomatic treatment of vertigo, nausea and vomiting, causative treatment to improve the recovery of vestibular function and improvement of central compensation. 50% questioned in our survey reported using antiemetics. This is what we refer to as symptomatic treatment. The efficacy has been proven by many clinical trials (21, 22). A limited use is recommended though as symptomatic treatment can delay vestibular compensation (23). The use of glucocorticoids was reported by 59.8% in our survey. Corticosteroids are counted as causative treatment due to their positive effect on vestibular function recovery. This has been shown by several studies, reviews and trials (24–27). In our survey, corticosteroids were administered for 1–7 days. This is in line with the recommendation of a meta-analysis conducted in 2021, which came to the conclusion that there is only a positive effect of glucocorticoids in the acute phase (28). Short-term application of corticosteroids in AUVP is also recommended in a recent guideline for reasonable practice in the emergency department (29). On the contrary, there is one meta-analysis which came to the conclusion that long-term application of corticosteroids could improve peripheral vestibular function and improve the long-term recovery (30). The third treatment principle in AUVP is improving central compensation either by vestibular physical therapy or pharmacotherapy (e.g., betahistine). According to our survey, 30% reported the application of betahistine in AUVP, although to date there is only questionable evidence for its efficacy (31, 32). The most important principle to re-establish central compensation consists of vestibular physical therapy (33, 34) (35), particularly in combination with corticosteroids, a beneficial effect was observed (36, 37). In our survey, 40% reported a combination of vestibular physical therapy and glucocorticoids, while 67% only applied vestibular physical therapy. Vestibular physical therapy is supported by a 2015 Cochrane review stating moderate to strong evidence for the efficacy and beneficial effects of vestibular rehabilitation (11).

To conclude, there is good evidence for the efficacy of vestibular physical therapy in AUVP improving central compensation. Also, the efficacy of symptomatic treatment has been proved by many clinical trials. In terms of causative management of AUVP, concise and standardized therapeutic guidelines are missing. Firstly, RCTs are warranted to establish standardized recommendations on glucocorticoid dosage and duration of application. Secondly, the role of betahistine and other drugs (e.g., N-acetyl-DL-leucine and the Ginko biloba extract) in improving and accelerating central vestibular compensation must be evaluated by RCTs.

### 4.3 Benign paroxysmal positional vertigo

In our world-wide survey, vestibular physical therapy was the main treatment strategy regardless of the affected canal. The use of canalith repositioning maneuvers is widely standardized and the first-line therapy.

In posterior canal benign paroxysmal positional vertigo (pc BPPV), almost all the respondents used the Epley maneuver (91.2%) followed by the Semont-maneuver (51.4%). This is in line with current recommendations describing the Semont, the SemontPLUS and the Epley maneuvers as gold standard in BPPV (38, 39). Several RCTs and meta-analysis (40–46) proved that the Epley maneuver is effective. Regarding the efficacy of the Semont maneuver, the current data situation is poorer, still the conducted trials indicate a beneficial effect (47–49). A meta-analysis comparing the efficacy and safety of the Epley and Semont maneuvers concluded that both maneuvers are equally effective and safe (50). A recently conducted clinical trial showed that the SemontPLUS maneuver is superior to the Epley maneuver and superior to the conventional Semont maneuver (51). In our survey, the questionnaire did not differentiate between Semont and SemontPLUS so that unfortunately no statement can be made regarding the frequency of use. A pharmacotherapeutic approach was used by 15% of the participants. Betahistine (58%), antiemetics (27.8%) and vitamin D (11.1%) were among the most frequently used oral drugs. Antiemetics are effective in the acute, symptomatic treatment for all described forms of BPPV (52) and commonly applied shortly before performing canalith repositioning maneuvers. The role of vitamin D deficiency as well as osteoporosis has been highly debated over the last years (53–56). A 2014 systematic review highlighted this topic for the first time, indicating that osteoporosis incidence in patients with BPPV is higher compared to controls (57). Additionally, a study conducted in 2015 found a seasonal variation (higher prevalence from December to May) with a negative correlation to the serum vitamin D concentration (58). Several studies have supported the vitamin D deficiency hypothesis (53, 59) while there have been others suggesting a coincidental existence (60). Betahistine has only been shown to be effective as add-on-therapy to canalith repositioning maneuvers (6, 39, 61, 62). 5% of our participants indicated using psychotherapy, in particular CBT. A randomized controlled study showed a beneficial effect of CBT combined with low-dose betahistine in treating BPPV (63). Still, more data is needed to demonstrate a beneficial effect. Surgery was only reported by 9%, mainly plugging of the posterior semicircular canal was used. A meta-analysis pointed out that although surgery is highly effective, there are potential serious complications such as deafness and loss of vestibular function (64).

In horizontal canal benign paroxysmal positional vertigo, we differentiated between canalolithiasis (hcBPPV-can) and cupulolithiasis (hcBPPV-cup). In hcBPPV-can, the Lempert (roll-over) maneuver was the most frequent canalith repositioning maneuver used among the participants (78.2%), followed by Gufoni maneuver (44.3%) and forced prolonged positioning maneuver (Vanucchi). The effectiveness of the Lempert (roll-over) maneuver has been shown in a 2012 RCT (65) but also Vanucchi has been proved to be effective (66). The advantage of Gufoni is that it can be applied to treat patients with hcBPPV-can and hc-BPPV-cup (65). This is reflected by our survey in which Gufoni was the most frequently used canalith repositioning maneuver in hc-BPPV-cup. Other used maneuvers were the Zuma maneuver, the head shaking maneuver, and analogous to hcBPPV-can, the Lempert (roll-over) maneuver. A comparison of head shaking only, Gufoni maneuver, and the Lempert (roll-over) maneuver showed no difference in the efficacy (67). In our survey, pharmacotherapy was used by 14% comprising betahistine (hcBPPV-can 66.7%, hcBPPV-cup 69.4%), antiemetics (hcBPPV-can 18.2%, hcBPPV-cup 22.2%), herbal medication (hcBPPV-can 18.2%, hcBPPV-cup 11.1%) and vitamin D (hcBPPV-can 12.1%, hcBPPV-cup 11.1%). Canal occlusion was reported by 3.8%, psychotherapy by none.

Anterior canal benign paroxysmal positional vertigo (ac BPPV) was mainly treated by the Yacovino maneuver (62.4%) and the modified Epley maneuver (24.1%). Theoretical 3D-simulations showed the superiority of the modified Yacovino maneuver to treat ac BPPV to the reverse Epley maneuver (68). Clinical validation studies are mandatory to prove this superiority outside of the 3D simulation. Only 10% reported using pharmacotherapy, betahistine was the most frequently reported drug (60.9%).

In conclusion, canalith repositioning maneuvers are state of the art and first line therapy in BPPV. Pharmacological add-on therapy might be beneficial, the current data situation remains poor and partially ambivalent though. A potential role of vitamin D deficiency still has to be evaluated by further RCTs. Due to the potential complications, semicircular plugging should only be considered in intractable cases.

### 4.4 Bilateral vestibulopathy

According to our survey, the main treatment strategy in BVP has been vestibular physical therapy (77%) with vestibular rehabilitation in the form of balance training. These measures are known to be effective (6, 69).

We received four reports of centers using vestibular implants. This small number is possibly explained by the fact that the vestibular implants are still undergoing further development and optimization (70).

In conclusion, apart from informing and educating the patient, vestibular physical therapy remains the most important treatment currently. Vestibular implants could be a promising treatment alternative, RCTs are warranted to evaluate their efficacy and safety in BVP,

### 4.5 Vestibular paroxysmia

The main treatment approach in our survey was pharmaceutical (65%), different antiseizure medication (ASM) have been used by our participants. There is various evidence from randomized controlled trials of a significant therapeutic efficacy of oxcarbazepine and carbamazepine (71–74). Lacosamide has been proven to be effective as well and is usually better tolerated (72) with fewer side effects than carbamazepine and oxcarbazepine (75). Other sodium channel blockers such as gabapentine, lamotrigine or topiramate are described as considerable options due to intolerance of carbamazepine or oxcarbazepine (74, 76). Nine participants (77.3%) reported treatment by surgery, Janetta decompression has been reported to be the most used surgical approach in our survey. Although there are reports on partial successes (77, 78), this approach should be considered as a last option due to the unfavorable benefit–risk-ratio (73).

To conclude, ASM such as oxcarbazepine and carbamazepine are effective and recommended treatment options. A better tolerated option may be the use of lacosamide. The Jannetta decompression appears to be an effective treatment in intractable cases, possible risks should be considered though. Measures such as vestibular physical therapy, psychotherapy and betahistine are currently considered to be of secondary importance, further clinical studies will be needed to evaluate a potential benefit.

### 4.6 Superior canal dehiscence syndrome

The preferred treatment among those we surveyed was surgery. The main surgical procedures in our survey were canal plugging (38.8%), capping (23.3%) and resurfacing (15.5%). So far there is no consensus on the best surgical method (79, 80). Meta-analysis and systematic reviews demonstrated that plugging and capping techniques are more successful than resurfacing (81, 82).Our questionnaire did not ask separately about conservative measures, so no statement can be made here.

To sum up, well-established surgical techniques, such as plugging, resurfacing, and capping, are currently used, although the therapy of choice is a conservative approach (6). Whether a conservative approach via vestibular physical therapy or betahistine would be beneficial still has to be evaluated.

## 5 Limitations

Within this study, limitations can be found. There is a bias because anybody with access to the survey could have answered anything without evidence of correctness (e.g., the reports for surgery in MD). Additionally, due to multiple answers in the world-wide survey, the respondents could have mentioned all interventions available without ever having used them. Furthermore, the reporting therapists each have different experience in treating peripheral vestibular disorders. They each see a different number of patients and most likely different severity in symptoms. Some therapists are in more experienced centers than others, e.g., working in private practice. This appears to be one reason for the heterogeneous replies.

Nonetheless, concerning the respondents’ qualifications, the survey targeted clinicians with expertise in peripheral vestibular disorders. Participants were recruited through internationally recognized institutions and societies, which served as proxies for professional credibility. Additionally, the survey included questions designed to verify clinical experience, such as the number of years practicing in the field and the frequency of vestibular disorder cases managed.

Although invitations were sent to participants from all countries in the world, not all centers, clinics, or physicians responded to the survey, which resulted in further limitations. Firstly, this snowball approach may have facilitated access to experts already connected within the networks of the Department of Neurology and German Center for Vertigo and Balance Disorders of Ludwig-Maximilians University Munich as well as the Bárány Society, potentially limiting the inclusion of a further extended pool of participants. Secondly, there was underrepresentation in certain regions, such as Africa and Oceania, due to their low number of reports compared to the number of reports from Europe, Asia, and the Americas. This disparity reflects the limited presence of experts in peripheral vestibular in different regions of the world. Consequently, these regions do not generate a significant number of responses, as the density of professionals with expertise in this area remains comparatively low. Nevertheless, the 234 gathered responses from five continents, 47 countries, 162 cities and 188 centers still are a representative sample size.

## Data Availability

The raw data supporting the conclusions of this article will be made available by the authors, without undue reservation.

## Footnotes

^1^ https://www.soscisurvey.de
